# Cooperative folding of intrinsically disordered domains drives assembly of a strong elongated protein

**DOI:** 10.1038/ncomms8271

**Published:** 2015-06-01

**Authors:** Dominika T. Gruszka, Fiona Whelan, Oliver E. Farrance, Herman K. H. Fung, Emanuele Paci, Cy M. Jeffries, Dmitri I. Svergun, Clair Baldock, Christoph G. Baumann, David J. Brockwell, Jennifer R. Potts, Jane Clarke

**Affiliations:** 1 Department of Chemistry, University of Cambridge, Lensfield Road, Cambridge CB2 1EW, UK; 2 Department of Biology, University of York, Wentworth Way, York YO10 5DD, UK; 3 Astbury Centre for Structural Molecular Biology, University of Leeds, Leeds LS2 9JT, UK; 4 European Molecular Biology Laboratory, Hamburg Unit, Notkestrasse 85, 22603 Hamburg, Germany; 5 Faculty of Life Sciences, Wellcome Trust Centre for Cell-Matrix Research, University of Manchester, Michael Smith Building, Greater Manchester M13 9PT, UK

## Abstract

Bacteria exploit surface proteins to adhere to other bacteria, surfaces and host cells. Such proteins need to project away from the bacterial surface and resist significant mechanical forces. SasG is a protein that forms extended fibrils on the surface of *Staphylococcus aureus* and promotes host adherence and biofilm formation. Here we show that although monomeric and lacking covalent cross-links, SasG maintains a highly extended conformation in solution. This extension is mediated through obligate folding cooperativity of the intrinsically disordered E domains that couple non-adjacent G5 domains thermodynamically, forming interfaces that are more stable than the domains themselves. Thus, counterintuitively, the elongation of the protein appears to be dependent on the inherent instability of its domains. The remarkable mechanical strength of SasG arises from tandemly arrayed ‘clamp' motifs within the folded domains. Our findings reveal an elegant minimal solution for the assembly of monomeric mechano-resistant tethers of variable length.

## Abstract (web summary)

Staphylococcal biofilm formation is promoted by the surface protein SasG. Here, the authors characterize the structure and remarkable mechanical strength of the repeat region of SasG, and show how elongation is achieved by obligate folding of the disordered regions within the repeating units.

## Graphical abstract

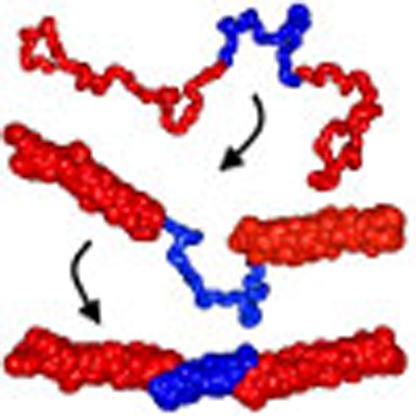

Bacteria adhere to surfaces, host cells and one another via specialized surface proteins (for example, pili, fimbriae or cellulosomes), which need to project away from the bacterial surface and be resistant to mechanical stress^1^,^2^. The mechanisms commonly used by bacterial adhesins to achieve both extension and mechanical strength are multimeric assembly and/or covalent stabilization^1^,^3^,^4^,^5^,^6^. SasG from *Staphylococcus aureus* and accumulation-associated protein from *S. epidermidis* are homologous proteins that promote host colonization and biofilm formation^7^,^8^,^9^. Staphylococcal biofilms are clinically important functional micro-communities of bacteria^10^ that cause hospital-acquired infections^11^ and promote exchange of antibiotic resistance genes^12^, presenting a significant global challenge.

Both SasG and accumulation-associated protein contain a central region with a variable (strain dependent) number of 128 amino acid repeats. The proteins are covalently attached to the cell wall at their carboxy terminus and are visible, using electron microscopy, as thin, highly extended fibrils on the cell surface (53–160 nm; strain dependent)^7^,^13^. Many bacterial surface fibrils, such as pili, are multi-chain assemblies^1^,^4^,^14^,^15^, which can have inter- and/or intramolecular covalent cross-links to maintain strong, highly extended structures^5^,^16^,^17^. Monomeric proteins that are formed from α-helical bundles can provide length^18^ but are intrinsically mechanically weak^19^. Alternatively, monomeric proteins with tandemly arrayed β-sandwich domains (for example, titin and fibronectin) have been shown to be mechanically strong^20^,^21^, but these do not form highly elongated structures^22^,^23^. The tandem repeats of SasG fold into two structurally related domains: E (50 residues) and G5 (78 residues), formed from single-layer triple-stranded β-sheets, framing a central collagen-like triple-helical region^24^,^25^ (Fig. 1). Interestingly, the E domain is disordered both in isolation and when preceded by a folded G5 domain (G5-E), but folded in E-G5 and G5-E-G5 to form elongated structures^24^. Whether and how such tandemly repeated, relatively unstable, β-sheet domains could underpin an elongated and mechanically strong structure is unclear.

Here we use a multidisciplinary approach, combining structural, computational and biophysical methods to demonstrate that the repeat region of SasG maintains a monomeric and highly extended conformation in solution, which is resistant to significant mechanical stress. The length is achieved by obligate folding of intrinsically disordered E domains to form stable interfaces that couple non-adjacent G5 domains and promote long-range cooperativity. The mechanical strength of SasG arises from tandemly arrayed ‘clamp' motifs within the folded G5 and E domains, formed from directly hydrogen-bonded β-strands. Our study reveals the molecular basis for the efficient formation of elongated and mechanically resistant bacterial adhesins from a single polypeptide chain.

## Results

### SasG is an extended monomeric molecule

**Figure 2:**
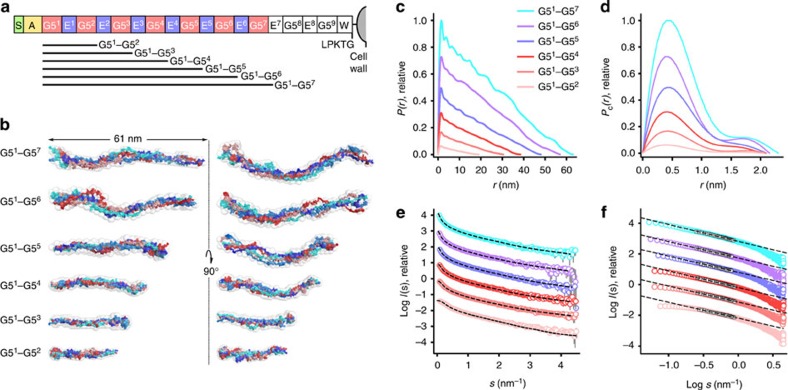
SAXS analysis of SasG particle shape and size. (**a**) Schematic of SasG in *S. aureus* strain NCTC 8325-4; signal sequence (S; cleaved), adhesion domain (A), E-G5 repeats, C-terminal wall (W) region and LPKTG signal for cell wall attachment; expression constructs are illustrated below. (**b–f**) SAXS studies of G5^1^–G5^2^, G5^1^–G5^3^, G5^1^–G5^4^, G5^1^–G5^5^, G5^1^–G5^6^ and G5^1^–G5^7^ (colour legend defined in **c**): (**b**) five *ab initio* bead models and their filtered average (grey); (**c**) distance distribution functions *P*(*r*) and (**d**) cross-sectional distance distribution functions *P*_*c*_(*r*) (for presentation purposes, *P*(*r*) and *P*_*c*_(*r*) have been scaled relative to construct length); (**e**) SAXS profiles (offset on log scale) and calculated scattering fits (black dashed lines) for: the G5^1^–G5^2^ X-ray crystal structure and a representative Gasbor *ab initio* model of all other constructs (see Supplementary Table 2 for *χ*^2^*-*values). (**f**) Porod exponents (Log *I*(*s*) versus Log *s* offset on log scale; linear regression analysis (black dashed lines)) fitted to the mid-*s* data range (white squares with black outline).

Only SasG with five or more E-G5 repeats promotes biofilm formation, likely to be due to the need to project above the layer of other cell surface components^7^. To generate a model for the tandemly arrayed repeats, we first determined the structure of G5^2^-E-G5^3^ (Supplementary Fig. 1a–h, Supplementary Table 1 and Supplementary Discussion), which has the highest sequence identity to other repeats. A model of contiguous repeats comprising all domains from G5^1^ to G5^7^ (generated by iterative superposition) had a length of 71 nm (Supplementary Fig. 1i). To assess the validity of this model experimentally, we produced constructs starting from G5^1^-E-G5^2^ with E-G5 increments up to G5^1^–G5^7^ (Fig. 2a); all were monomeric and monodisperse (Supplementary Fig. 2a and Supplementary Table 2). We determined their shape in solution using small-angle X-ray scattering (SAXS) (Fig. 2b–f, Supplementary Fig. 2b–g and Supplementary Discussion). The maximum particle dimension, *D*_max_ of G5^1^-E-G5^2^ matched the crystal structure (19 nm; Fig. 2c) and the calculated scattering matched the experimental SAXS data (*χ*^2^=1.12; Fig. 2e and Supplementary Fig. 2f). The *D*_max_ increased incrementally with stepwise addition of E-G5 units (Fig. 2b,c). The *D*_max_ of 63 nm for G5^1^–G5^7^ is only 8 nm (∼11%) shorter than our (maximally extended) crystal structure-based model (Supplementary Fig. 1i). At the same time, the maximum particle cross-section (Fig. 2d and Supplementary Table 2) remains nearly unchanged and displays only a minor increase from 2.0 nm for G5^1^–G5^2^ to 2.3 nm for G5^1^–G5^7^. SAXS *ab initio* models (Fig. 2b) suggest that although highly extended on average, the particles may slightly coil or bend, which explains the observed increase in the effective cross-section.

**Figure 3:**
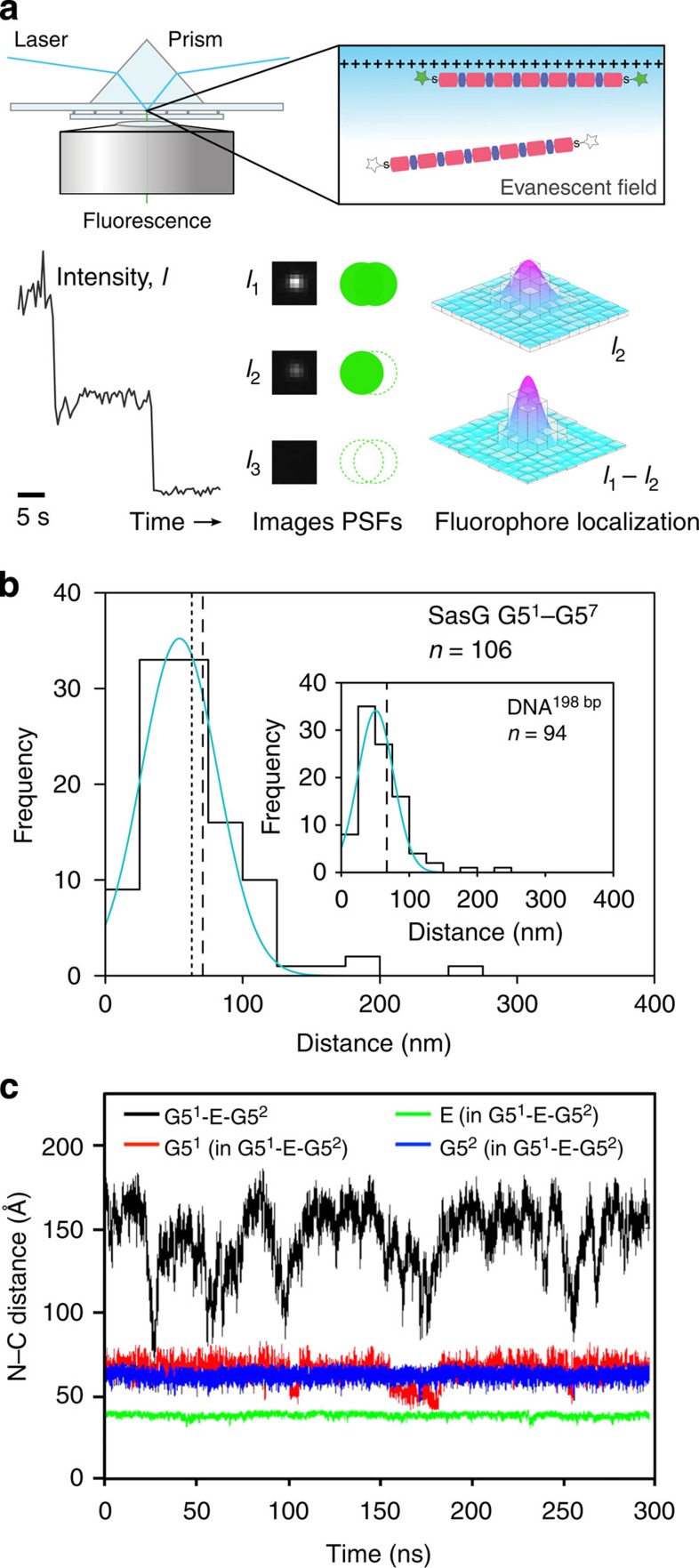
Conformational flexibility of SasG. (**a**) SHRImP-TIRFM experimental method: Fluorophores were attached to the N- and C-termini of G5^1^–G5^7^ via engineered cysteine residues. Fluorescently labelled protein was immobilized on a quartz slide surface using poly-D-lysine and visualized by prism-coupled TIRFM. By stepwise fluorophore photobleaching, individual point spread functions (PSFs) were analysed and interfluorophore distances calculated. (**b**) Interfluorophore distances for Alexa Fluor 488-labelled G5^1^–G5^7^ and 198 bp DNA (inset) on a poly-D-lysine (100 μg ml^−1^)-treated quartz surface. Solid lines indicate Gaussian fits to the histograms (mean=54 and 51 nm, respectively). Dashed lines indicate end-to-end distances based on crystallographic data for G5^1^–G5^7^ and B-form DNA (71 nm (Supplementary Fig. 1i) and 67 nm, respectively); the dotted line indicates the SAXS determined *D*_max_. (**c**) All-atom MD simulations of G5^1^-E-G5^2^. The N-to-C distance as a function of time is plotted for G5^1^-E-G5^2^ (black) and individual domains within the protein (G5^1^—red, E—green and G5^2^—blue). G5^1^-E-G5^2^ most frequently adopts an extended conformation (with the length of ∼170 Å, as observed in the crystal structure), but rare bent conformations (Supplementary Fig. 4b) are also sampled at room temperature (N-to-C distance fluctuates between 75 and 180 Å). As the individual domains maintain their fully extended crystallographic conformations throughout the simulation, the observed flexibility of G5^1^-E-G5^2^ appears due to bending at the G5-E and E-G5 interfaces (Supplementary Fig. 4).

To further corroborate the SAXS results, we adapted a high-resolution single-molecule imaging technique (SHRImP^26^; Fig. 3a and Supplementary Discussion). G5^1^–G5^7^, labelled with fluorophores at both ends, was visualized using total internal reflection fluorescence microscopy (TIRFM) and exhibited end-to-end distances consistent with the SAXS analysis (mean±s.e.=59±5 nm, Fig. 3b and Supplementary Fig. 3a,b). As this is the first reported application of SHRImP to the measurement of an end-to-end distance in an elongated protein, a control experiment using DNA of similar predicted length was performed (Fig. 3b inset and Supplementary Fig. 3a,b inset). All-atom molecular dynamics (MD) simulations of G5^1^-E-G5^2^ show that the individual G5 and E domains are relatively rigid and do not bend (Fig. 3c and Supplementary Fig. 4a). Analysis of the trajectories suggests that the collagen-like triple helix region provides rigidity within the domains (Supplementary Fig. 4b). Flexing of the molecule occurs almost exclusively at the interfaces between domains, which nonetheless maintain an overall linear profile (Fig. 3c and Supplementary Fig. 4), explaining the small difference between the rigid modelling from the crystal structure (Supplementary Fig. 1i) and the solution SAXS-based model (Fig. 2b). Thus, SasG is monomeric and extended in solution on length scales consistent with the extended fibrils observed on the bacterial cell surface.

### SasG behaves as series of overlapping cooperative units

**Figure 4:**
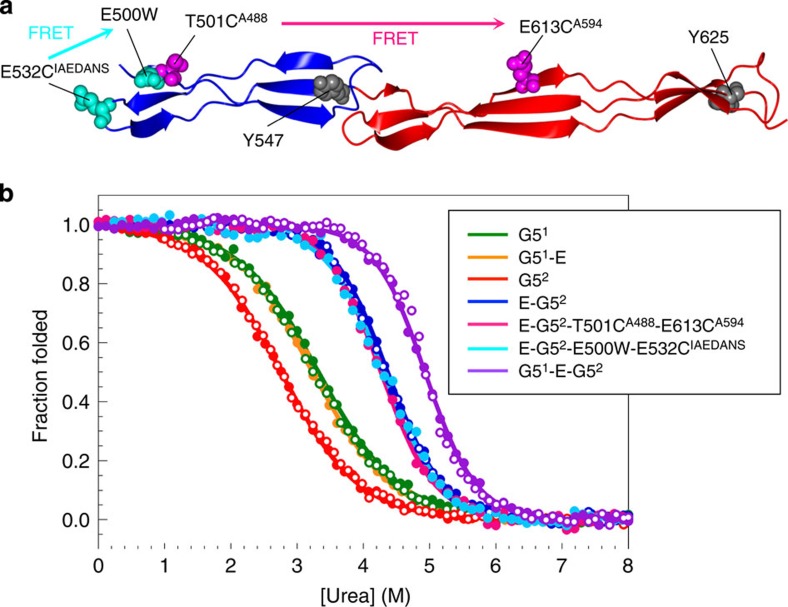
Equilibrium denaturation curves for SasG domains. (**a**) Structure of the two-domain fragment of SasG—E-G5^2^. E and G5^2^ domains are shown in blue and red, respectively. The location of tyrosines and residues used to engineer the FRET pairs are indicated and colour-coded. E and G5 domains each contain a single tyrosine residue (grey) located in the last strand of the C-terminal β-sheet. FRET pair T501C^A488^-E613C^A594^ (magenta) results in FRET when both E and G5^2^ domains are folded and was used to monitor (un)folding of E-G5^2^. FRET pair E500W-E532C^IAEDANS^ (cyan) results in FRET when the E domain is folded and was used to monitor (un)folding of E in the context of E-G5^2^. (**b**) Equilibrium unfolding (closed circles) and refolding (open circles) data for wild-type G5^1^ (green), G5^1^-E (orange), G5^2^ (red), E-G5^2^ (blue) and G5^1^-E-G5^2^ (purple), as well as E-G5^2^-T501C^A488^ -E613C^A594^ (magenta) and E-G5^2^-E500W-E532C^IAEDANS^ (cyan). Data for the wild-type proteins were collected by monitoring the change in intrinsic tyrosine fluorescence, whereas the FRET signal was measured as the change in acceptor fluorescence (Alexa Fluor 594 and 1,5-IAEDANS). All data were fit to a two-state model of unfolding (see Table 1 for thermodynamic parameters).

To determine the molecular mechanism whereby SasG maintains an extended conformation, we investigated the stability and folding kinetics of G5^2^ in isolation and in tandem with E (E-G5^2^). The unfolding was monitored by both intrinsic tyrosine fluorescence and specific FRET (Förster resonance energy transfer) measurements (Fig. 4). Irrespective of the method used, equilibrium unfolding for both G5^2^ and E-G5^2^ was reversible and characterized by a single transition, indicating two-state, all-or-none unfolding for both constructs (Fig. 4). We did not observe any separate folding or unfolding events when specific FRET probes were used to monitor the folding of only E or G5^2^ in the context of E-G5^2^ (Fig. 4 and Supplementary Fig. 5). E-G5^2^ is more stable than G5^2^ alone, by ∼3.5 kcal mol^−1^ (Table 1). Although E remains unfolded^24^ in G5^1^-E, once folded cooperatively with G5^2^, E interacts with and stabilizes the G5^1^ domain such that G5^1^-E-G5^2^ unfolds at even higher concentrations of denaturant and again in an apparently cooperative manner (Fig. 4b and Table 1). Hence, each G5 repeat is stabilized by the folding of the following G5, even though they are not in direct contact. The instability of E is required for the long-range cooperativity between G5 domains; this cooperativity would not exist if the E domains folded independently. The entire repeat region can be considered as a series of overlapping G5-E-G5 cooperative units (Fig. 2a) and thus will form a single cooperative unit.

### Long-range cooperativity is mediated by intrinsic disorder

In all-β tandem repeat proteins such as titin, with immunoglobulin-like domains, the individual domains are significantly more stable than the domains in SasG, but the domains do not fold cooperatively^27^ and the proteins are not fully elongated in solution^22^,^28^. The cooperativity in SasG is mediated by the intrinsic instability of the E repeats and by the relative stability of the interfaces. As explained in more detail in the Supplementary Discussion, we can estimate minimal stabilities conferred by the interfaces. The G5^1^-E and E-G5^2^ interfaces must confer at least 1.5 and 6 kcal mol^−1^, respectively, compared with the stabilities of the individual domains G5^1^, E and G5^2^ (−3.2, ≥+2.5 and −2.8 kcal mol^−1^, respectively). Herein lies the explanation for the remarkable elongation of the SasG molecule. In most tandem domain protein systems, the ‘weak link' is the inter-domain interface. In SasG, the interfaces provide more stability than the domains themselves and are maintained even in the most flexed structures in the MD simulations (Supplementary Fig. 4b). It is these interfaces, formed on the folding of E, that mediate the long-range cooperativity.

The role of intrinsic disorder in promoting folding cooperativity^29^ and allosteric coupling^30^,^31^ has been reported for other systems. In multi-domain proteins, where all domains are folded, cooperativity is short range and limited strictly to neighbouring domains; as has been clearly demonstrated for spectrin domains, domain *i* will not be affected by domain *i+2* if domain *i+1* is stable and folded^32^. At the other extreme, repeat proteins, such as proteins with helical ankyrin repeats, behave cooperatively, because the individual units are highly unstable and the linking interfaces are strong^29^. The SasG system is an intermediate case: the regions of disorder (E domains) are interspersed with stably folded G5 domains, but the high stability of the G5-E and E-G5 interfaces (relative to inherent instability of E) induces cooperativity between non-adjacent G5 domains. Thus, when G5^2^ folds, this induces the folding of E and facilitates formation of the G5^1^-E interface, which imparts stability to G5^1^.

### SasG has remarkable mechanical strength

**Figure 5:**
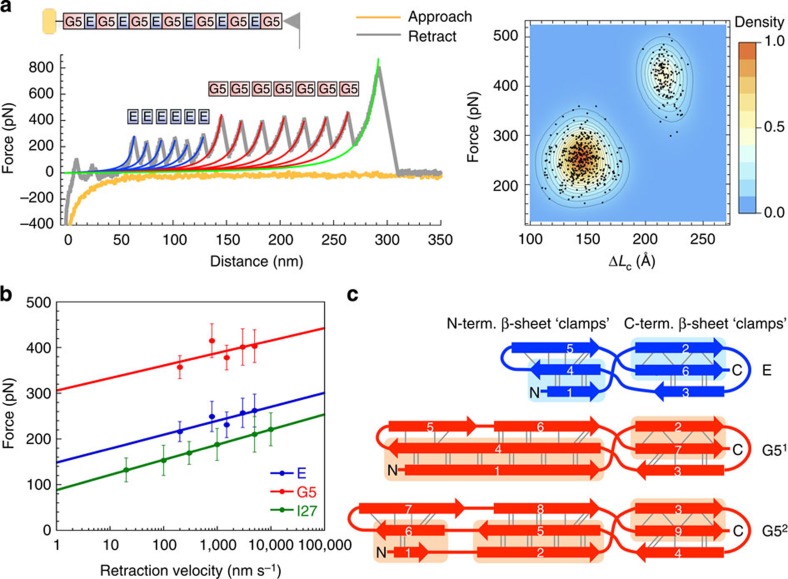
Mechanical resistance of SasG domains probed by AFM. (**a**) A sample AFM force-extension profile for a SasG construct containing seven G5 and six E domains (G5^1^–G5^7^; grey trace). Schematic of the experimental setup and the correlating scatter plot superposed with a smooth density histogram are also shown. The yellow trace represents the AFM cantilever tip approaching the surface. The six lower-force unfolding peaks (around 250 pN at a retraction rate of 800 nm s^−1^; labelled and indicated in blue) are associated with a contour length change (Δ*L*_c_) of 145 Å. The difference in length between a folded and unfolded E is ∼143 Å and these events thus correspond to unfolding of E domains. The seven higher-force peaks (around 420 pN at a retraction rate of 800 nm s^−1^; labeled and indicated in red) relate to a Δ*L*_c_ of 216 Å, and hence represent unfolding of G5 domains (the difference in length between a folded and unfolded G5 is ∼213 Å). The last force peak (indicated in green) represents the detachment of the protein from the tip or the surface. (**b**) Plot of mode unfolding forces (three independent experiments were performed for each pulling speed; see Supplementary Fig. 6 and Table 2 for details) against retraction velocity for SasG domains (E shown in blue and G5 in red) and I27 (shown in green; data taken from Best *et al.*^70^). Error bars represent s.d. E, G5 and I27 show a similar dependence of unfolding force on pulling speed, but SasG domains are mechanically more stable than I27. (**c**) Topology diagrams of SasG domains (E, G5^1^ and G5^2^) with ‘mechanical clamps' indicated as shaded boxes. Hydrogen bonds are shown as dotted lines. As revealed by MD simulations (Supplementary Fig. 7), the remarkable mechanical strength of SasG domains originates from tandemly arrayed mechanical clamps involving long stretches of hydrogen bonds and associated side-chain packing interactions along the β-strands.

SasG is anchored to the cell wall at the C-terminus and to other cells via its N-terminus^9^; thus, SasG molecules are likely to experience longitudinal mechanical stress *in vivo*. We used atomic force microscopy (AFM) to measure the mechanical strength of SasG G5^1^–G5^7^ (Fig. 5a). Approach-retract cycles were performed at various retraction velocities (200, 800, 1,500, 3,000 and 5,000 nm s^−1^), recording the force as a function of extension (Fig. 5a,b, Supplementary Fig. 6 and Table 2). Contrary to chemical denaturation, under force, SasG domains unfold independently of each other. Irrespective of the pulling speed, two types of unfolding peaks were observed: lower- and higher-force peaks (Supplementary Fig. 6). The lower-force unfolding peaks are associated with a contour length gain (Δ*L*_c_) of ∼150 Å (ranges from 145 to 154 Å for different retraction rates; Supplementary Fig. 6 and Table 2) and, as the difference in length between folded and unfolded E is ∼143 Å, these events correspond to unfolding of E domains. The difference in length between folded and fully extended G5 is ∼213 Å; hence, the higher-force peaks associated with Δ*L*_c_ of ∼220 Å (ranges from 216 to 227 Å for different retraction rates; Supplementary Fig. 6 and Table 2) represent unfolding of G5 domains. SasG domains show remarkable mechanical strength (Fig. 5b and Table 2). The weaker E domain, unstable in isolation, unfolds at forces of 250 pN (at 800 nm s^−1^ retraction rate), which is higher than needed to unfold the ‘strength-paradigm' 27th immunoglobulin domain from titin (I27; ∼180 pN)^33^, as well as other ‘strong' proteins^34^. The mechanical resistance of the G5 domain (420 pN at 800 nm s^−1^ pulling speed) falls into the upper limit of mechano-stability for any protein domain stabilized solely by non-covalent interactions^34^,^35^. Mechanical unfolding of SasG domains was also predicted by MD simulations revealing that the strength originates from tandemly arrayed ‘mechanical clamps'^34^ (Fig. 5c and Supplementary Fig. 7). Mechanical clamps are structural elements that determine the protein's primary resistance to tensile force; for example, the mechanical clamps of globular β-sandwich domains, such as I27, are formed by directly hydrogen-bonded N- and C-terminal β-strands. In the case of SasG, E and G5 domains contain an N-terminal β-sheet clamp, formed by two anti-parallel β-strands, and a C-terminal β-sheet clamp, composed of two parallel β-strands. Both N- and C-terminal clamps involve long stretches of hydrogen bonds and associated side-chain packing interactions along the β-strands (Fig. 5c and Supplementary Fig. 7). G5 domains have significantly longer N-terminal clamps than E domains, explaining the higher mechanical resistance of G5 relative to E.

Under mechanical force, the protein is no longer behaving as a cooperative unit. E and G5 domains unfold independently; the E domains act as force ‘buffers' relieving mechanical stress without complete unfolding of the SasG molecule. As the E domains fold rapidly when the G5 domains are folded, this allows for rapid recovery once stress is released. We speculate that SasG can oscillate between a ‘flexible' state under force, with uncoupled G5 domains and E unfolded, and a ‘stiff' state in the absence of force with all domains folded.

## Discussion

Nature can form elongated single-chain proteins or strong single-chain proteins. Here we have discovered how nature can form, from apparently insubstantial building blocks, a monomeric structure that is both long and strong. The length is maintained by using intrinsic disorder to form highly cooperative and stable interfaces that mediate communication between non-adjacent, stiff domains. Strength results from the optimal use of small domains that nonetheless contain long, aligned β-strands. Our findings provide a paradigm for efficient formation of a strong, highly elongated protein structure from a single polypeptide chain. Such protein rods of tunable length and requiring no additional covalent stabilization have significant potential for incorporation into novel biomaterials.

## Methods

### Chemicals

All chemicals were purchased from Sigma-Aldrich, unless otherwise stated.

### Protein production and characterization

Purification of short SasG repeats (G5^1^–G5^2^; residue range 420–629) was described previously^24^. A construct of G5^2^–G5^3^ (residue range 545–757) was expressed and purified similarly. The coding sequences of G5^1^–G5^3^ (residue range 419–757), G5^1^–G5^4^ (419–885), G5^1^–G5^5^ (419–1,013), G5^1^–G5^6^ (419–1,141) and G5^1^–G5^7^ (419–1,269) were synthesized by Genewiz and fused to an N-terminal hexahistidine-3C protease-cleavable tag and a non-cleavable C-terminal Strep-tag II (WSHPQFEK) in a modified pET28 vector, pET-YSBLIC (His_6_-3C-G5^1^-G5^x^–Strep). A second G5^1^–G5^7^ construct incorporated two cysteine residues at the C-terminus of the Strep affinity tag for AFM force unfolding studies generating His_6_-3C-G5^1^-G5^7^–Strep-CysCys using primer pair:

5′-TCCAGGGACCAGCAATGGCACCTAAGACCATCACC-3′

5′-TGAGGAGAAGGCGCGTTAGCAGCATTTTTCAAATTGAGGAT GAGACCAGGTCTCCGGACCATACTC-3′

A third G5^1^–G5^7^ construct was engineered into a XhoI/NdeI-digested pET26b(+) vector (Novagen), to generate a non-cleavable C-terminal His tag; cysteine residues replaced E418 and T1269 for SHRImP-TIRFM studies (Cys-G5^1^-G5^7^–Cys-His_6_) with primers:

5′-AAGAAGGAGATATACATATGGGTGGCTGCGCACCTAAGACCATCAC-3′

5′-GGTGGTGGTGCTCGAGGCCGCTGCTGCCGCACTCCGGACCATACTCGG-3′

Liquid cultures of LB supplemented with 50 μg ml^−1^ kanamycin were inoculated with transformed *Escherichia coli* BL21(DE3) cells (Novagen) and grown at 37 °C to OD_600_ 0.5–0.6; expression was induced with addition of 1 mM isopropyl-β-D-thiogalactopyranoside, followed by incubation at 20 °C for 20 h. Cells were resuspended in 500 mM NaCl, 20 mM imidazole, 20 mM Tris HCl, pH 7.5, supplemented with EDTA-free protease inhibitor cocktail (Roche) and lysed by sonication. Soluble extract was purified by nickel affinity chromatography. Constructs with 3C protease sites and Strep tags were digested with HRV-3C protease and purified by Strep-Trap affinity chromatography (GE Healthcare). Eluted protein was separated by size-exclusion chromatography (SEC) on a Superdex 200 26/60 column (GE Healthcare) in 200 mM NaCl, 1 mM EDTA and 20 mM Tris HCl, pH 7.5. His_6_-3C-G5^1^-G5^7^–Strep-CysCys and Cys-G5^1^-G5^7^–Cys-His_6_ were also purified by SEC with the addition of 2.5 mM dithiothreitol (DTT). Molecular masses were characterized by electrospray ionization mass spectrometry. SAXS samples were dialysed against SEC buffer to generate matched solvent for buffer scattering subtraction. Protein concentrations were estimated by A_280_. SEC-multi-angle laser light scattering–quasi-elastic light scattering analysis of samples in the range 1.5–2 mg ml^−1^ was performed using a Superdex 200 10/300 GL column (GE healthcare) in line with a Dawn HELEOS-II 18-angle light-scattering detector, Optilab rEX refractive index monitor and quasi-elastic light scattering detector (Wyatt).

### Crystallography

G5^2^–G5^3^ was concentrated to 26.2 mg ml^−1^ and screened for crystallization at 18 °C. Crystals grew in 3 days in JCSG-*plus* (Molecular Dimensions) condition D2 (0.2 M MgCl_2_, 0.1 M Na HEPES pH 7.5, 30% v/v PEG 400). Crystals were vitrified in liquid N_2_ and two data sets collected from a single crystal by offsetting the crystal by *κ*=40° using a mini-Kappa goniometer at Diamond light source beamline I04, wavelength 0.9795 Å at 100 K. Data were integrated using XDS^36^, merged and scaled using Aimless^37^, and molecular replacement performed using PhaserMR^38^. E^1^G5^2^ (PDB accession: 3TIP) was separated into two search models: E^1^ (residue range 502–548) and G5^2^ (residue range 549–629). The model was partially built using Buccaneer^39^, completed with Coot^40^ and refined with Refmac5 (ver. 5.8.0073)^41^. TLSMD server was used to define translation/libration/screw groups (residue ranges 543–588, 589–677 and 678–757)^42^. Aimless, PhaserMR, Buccaneer and Refmac5 were implemented through the CCP4 interface^43^. The final model was evaluated with MolProbity^44^ and the Ramachandran plot showed that all residues have favourable *ϕ*/*ψ* geometry. G5^1^–G5^2^ (PDB accession: 3TIQ) superposition was performed using Gesamt^45^. The G5^1^–G5^7^ particle was modelled by superposition of G5^1^–G5^2^ and G5^2^–G5^3^, and iterative superposition of G5^2^–G5^3^ by secondary structure matching^46^.

### Small-angle X-ray scattering

SAXS intensity data, *I*(*s*) versus *s*, (*s*=4*π*sin*θ*/*λ*, where 2*θ* is the scattering angle, 0.04–4.4 nm^−1^) were collected from protein samples and matched solvent blanks at the EMBL-P12 beamline at PETRAIII (DESY, Hamburg) employing automated data acquisition and radial averaging protocols^47^. Guinier (ln *I*(*s*) versus *s*^2^) and modified Guinier analysis for extracting cross-sectional terms (ln (*I*(*s*)*s*) versus *s*^2^) were performed using Primus^48^. Indirect inverse Fourier transformations of the data were calculated using GNOM^49^, to generate probable real-space atom-pair distance distributions (*P*(*r*) versus *r*) and distance distributions of cross-sections (*P*_*c*_(*r*)). Porod exponents were calculated from the slope of linear fits to the decay in scattering intensity of Log *I*(*s*) versus Log *s* through the mid-*s* regions of the profiles^50^,^51^. The molecular masses (MMs) were evaluated from the forward-scattering intensity *I*(*0*) placed on an absolute scale (cm^−1^) using the scattering from water^52^, combined with calculated values of concentration, partial specific volume and scattering-length density contrasts^53^ derived from amino acid sequence and atomic compositions. The MM was additionally calculated relative to the scattering calibrated using a BSA standard^54^ and SAXS.MoW was used to estimate the concentration-independent MM based on apparent volumes^55^. *Ab initio* shape restorations using a dummy residue algorithm Gasbor^56^ were performed five times against each SAXS data set, and aligned and averaged using Damaver^57^ to generate a consensus three-dimensional shape. The model fit of the X-ray crystal structure against the SAXS data was calculated using Crysol^58^. Rigid body modelling was performed using SASREF^59^.

### Production of fluorescently labelled protein

Cysteine residues were engineered into the N- and C termini of G5^1^–G5^7^ where quenching by nearby residues is least probable. The protein (50 μM) was incubated with 20 × molar excess of Alexa Fluor 488 C_5_ maleimide (Life Technologies) in 20 mM MOPS, 200 mM NaCl, 0.1 mM tris(2-carboxyethyl)phosphine, pH 7.0, at 25 °C for 2 h. The reaction was quenched by the addition of DTT to a final concentration of 5 mM. The protein was then dialysed into storage buffer (20 mM KH_2_PO_4_/K_2_HPO_4_, 200 mM NaCl, 1 mM DTT, pH 7.5) and purified by SEC on an S200 10/300 column (Amersham), to remove all remaining free dye before storage at −80 °C.

### Production of fluorescently labelled DNA

A 215-bp DNA fragment was PCR amplified from bacteriophage T7 DNA using fluorescently labelled primers

5′-CCCCAAGCTTCATCTZGTCAGATGAGACTACCCCTCTGAA-3′

and

5′-CCZCAAAGTCTGTACTTTTAGTAGGTCTTATAGTCC-3′

where ‘Z' indicates an internal Alexa Fluor 488-dT modification, such that the fluorophores are separated by 198 bp. An inter-fluorophore distance of 62–67 nm was predicted using the DNA curvature analysis tool ( http://www.lfd.uci.edu/~gohlke/dnacurve/) and model.it^60^, which takes into account sequence-dependent static bending.

### Sample preparation for TIRF microscopy

Poly-D-lysine-coated quartz slides were prepared by incubating the slides at 20–22 °C for 30 min in 20–500 μg ml^−1^ poly-D-lysine hydrobromide (30–70 kDa, Sigma), 10 mM MOPS, pH 7.0. The slides were then rinsed by dipping in deionized water and dried with filtered air. Alexa Fluor 488-labelled protein and DNA samples were prepared by diluting concentrated stocks into imaging buffer (10 mM HEPES, 10 mM NaCl, 5 μM β-mercaptoethanol, 1 mM Trolox, pH 7.0) with streptavidin-derivatized quantum dots emitting at 655 nm (100 pM, Quantum Dot Corporation). Five-micrometre silica beads (∼0.1 mg ml^−1^) were included in all samples, to specify the height of flow cells and to prevent coupling of excitation light into the coverslip. Flow cells were constructed by applying sample (25 μl) to the slide, covering with coverslip (No. 1, 22 mm × 64 mm, Menzel-Gläser) and sealing two opposite short sides with nail varnish. Ten minutes were allowed for the protein to immobilize. Any unbound material was subsequently washed out with two volumes of imaging buffer and the flow cell sealed with nail varnish.

### TIRF microscopy

Prism-coupled TIRFM was performed using a custom-modified inverted IM35 microscope (Carl Zeiss AG) at 20–22 °C. Fluorophores were excited with 488 and 561 nm lasers (Coherent) operated at 10 mW and 30–50 mW output, respectively. A 488-nm zero-order quarter-wave plate (Edmund Optics) was installed to circularly polarize the incident laser light, thus removing any dye orientation-dependent fluorescence excitation. The quantum dots were used as an image-focusing aid (with 561 nm laser illumination only), thus minimizing Alexa Fluor 488 dye photobleaching before video acquisition. Fluorescence emission was captured through a Plan-Apochromat × 100/numerical aperture 1.4 oil-immersion objective (Carl Zeiss AG). A dual-view image splitter (OptoSplit II, Cairn Research) with appropriate emission dichroic (580 nm long pass, Zeiss) and bandpass filters for the Alexa Fluor 488 dye (ET525/50M, Chroma) and quantum dots (ET605/70M, Chroma) was used to split the image into two fluorescence emission channels. Video data were collected using an Evolve 512 electron-multiplying CCD (charge-coupled device) camera (Photometrics), cooled to −70 °C and operated through μManager^61^ with 500 ms exposure (2 fps). Pixel size was equivalent to 157 nm in the magnified image as determined using a USAF calibration target (Edmund Optics).

### Detection and localization of single fluorophores

Fluorescent spots were detected using the multi-step test (using standardized full width at half maximum and intensity threshold values) in GMimPro^62^. Fluorophores that photobleached in two steps were manually selected and their *x*,*y* coordinates exported. Spot intensities for individual particles were then extracted in ImageJ^63^ as an image stack for a 10 × 10 pixel^2^ area. Subsequently in MATLAB (MathWorks, Cambridge, UK), the intensity profile of each particle was processed using a Chung-Kennedy filter^64^ and a derivative-based step detection method was implemented to identify the three intensity states of the particle: *I*_1_, two fluorophores fluorescing; *I*_2_, one fluorescing; *I*_3_, none fluorescing. Mean images of the seven frames before and after the first step were used to calculate *I*_1_ and *I*_2_, respectively. Fluorophore locations were determined by fitting a two-dimensional Gaussian function (with integration over each pixel) to *I*_2_ and the difference between *I*_1_ and *I*_2_:


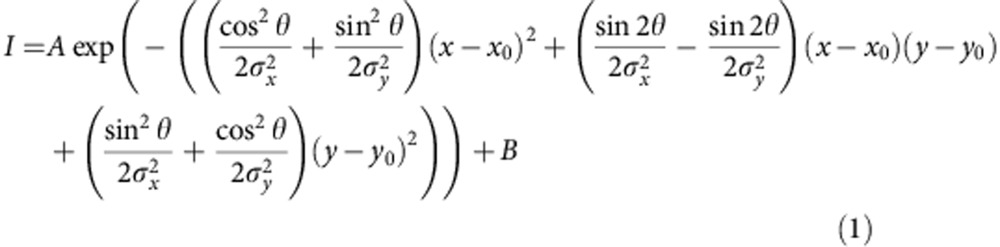


where *A* is amplitude with centre (*x*_0_, *y*_0_), *σ*_*x*_ and *σ*_*y*_ describe the widths, *θ* specifies rotation of the function about the centre and *B* accounts for background intensity. End-to-end distances were calculated for pairs of fluorophores with eccentricity <0.04 and presented as histograms. A bin size of 25 nm was chosen to satisfy the Freedman–Diaconis rule^65^. A Gaussian distribution of the form


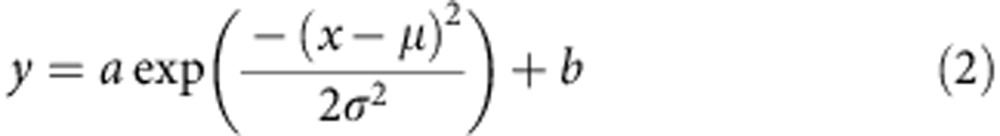


was fitted to each histogram.

### MD simulations

Simulations were performed using a united-atom force field (CHARMM19) and implicit solvent model (FACTS). All simulations were performed at 300 K, with Langevin dynamics using the leapfrog integrator, a timestep of 2 fs and a friction coefficient of 3 ps^−1^, and run using CHARMM. The CHARMM19 FACTS parameters used were: dielectric constant=2.0, nonpolar surface tension coefficient=0.015 kcal mol^−1^ Å^−2^. Within the FACTS implicit solvent, the influence of salt has been taken into account on the Debye-Hückel level^66^. The friction coefficient of 3 ps^−1^ corresponds to a viscosity about 20 times smaller than that of water, thus enhancing sampling efficiency by a corresponding factor, but it is sufficiently large to ensure that dynamics is diffusive and mechanisms observed independent on the friction coefficient^67^. Trajectory frames (and associated analysis parameters) were recorded every 500 steps. Simulation for G5^1^-E-G5^2^ was started from the crystal structure (PDB accession: 3TIQ) and continued for 930 ns. Forced unfolding was simulated by attaching an ideal spring to the N and C atoms of the two termini and retracting them at constant speed. Simulations were performed in conditions corresponding to monovalent salt concentration of both 0 and 200 mM (experiments were performed in solution containing between 140 and 200 mM NaCl). No significant differences were observed between low (0 mM) and higher salt (200 mM) conditions.

### FRET labelling

Tryptophan (E500W) and cysteine (T501C, E532C and E613C) residues were introduced by site-directed mutagenesis using the following primers:

E500W

5′–GTTCTGTTCCAGGGGCCCTGGACGATCGCGCCGGGTC-3′

5′-GACCCGGCGCGATCGTCCAGGGCCCCTGGAACAGAAC-3′

T501C

5′-GTTCCAGGGGCCCGAATGTATCGCGCCGGGTCACC-3′

5′-GGTGACCCGGCGCGATACATTCGGGCCCCTGGAAC-3′

E532C

5′-CCGGGTATCAAAAACCCGTGTACCGGTGACGTTGTTCGTCC-3′

5′–GGACGAACAACGTCACCGGTACACGGGTTTTTGATACCCGG-3′

E613C

5′-CATCTCTAAAGGTGAATCTAAAGAATGTATCACCAAAGACCCGATCAACGAAC-3′

5′-GTTCGTTGATCGGGTCTTTGGTGATACATTCTTTAGATTCACCTTTAGAGATG-3′

Labelling was carried out using thiol-reactive probes, according to manufacturer's procedures. Labelling of E-G5^2^-E500W-E532C was performed by mixing reduced protein with a 20-fold molar excess of 5-((((2-iodoacetyl)amino)ethyl)amino)naphthalene-1-sulfonic acid (1,5-IAEDANS; Life Technologies; acceptor). The labelling reaction proceeded for ∼14 h at 4 °C. Unreacted dye was removed by gel filtration (HiTrap Desalting column; GE Healthcare) and the degree of labelling was estimated as ∼100%. Labelling of E-G5^2^-T501C-E613C was carried out using Alexa Fluor 488 (donor) and Alexa Fluor 594 (acceptor) maleimides (Life Technologies). The dyes were added to reduced protein simultaneously in equimolar ratios and incubated at 4 °C for ∼14 h. Unreacted dyes were removed by gel filtration (HiTrap Desalting column; GE Healthcare) and the hetero-doubly labelled protein (E-G5^2^-T501C^A488/594^-E613C^A488/594^) was isolated on a HiTrapQ HP column (GE Healthcare).

### Equilibrium studies

The free energy of unfolding of the proteins was determined by chemical denaturation using urea. Fluorescence measurements were carried out on a Perkin Elmer LS55 fluorescence spectrometer under standard conditions (PBS, 25 °C). The samples were equilibrated for at least 2 h before data collection. In the intrinsic tyrosine fluorescence studies, the protein concentration was 5 μM, the excitation wavelength used was 276 nm and the emission was followed at 305 nm. In the case of E-G5^2^-E500W-E532C^IAEDANS^, the protein concentration was 500 nM, the excitation wavelength was 280 nm (tryptophan excitation) and the emission was followed at 493 nm (IAEDANS fluorescence). The data for E-G5^2^-T501C^A488^-E613C^A594^ were collected at a protein concentration of 50 nM, the excitation wavelength was 495 nm (Alexa Fluor 495 excitation) and the emission was followed at 612 nm (Alexa Fluor 594 fluorescence). The data were fit to a standard two-state equation.

### Kinetic studies

The kinetic experiments monitoring fluorescence change were carried out using an Applied Photophysics SX.20 stopped-flow fluorimeter maintained at a temperature of 25 °C. The final protein concentration and the excitation wavelength used were the same as described for equilibrium studies. No cut-off filter was used in the experiments for wild-type proteins monitoring the change in tyrosine fluorescence. Cut-off filters (435 and 590 nm) were used to collect the data for E-G5^2^-E500W-E532C^IAEDANS^ and E-G5^2^-T501C^A488/594^-E613C^A488/594^, respectively. At least 20 traces were averaged for a typical measurement at a given urea concentration. Kinetic traces were analyzed using Kaleidagraph 4.1.3 (Synergy Software). All rate constants were independent of protein concentration under the experimental conditions. Chevron plots (the dependence of the logarithm of the observed rate constant on the concentration of urea) were fit either to a standard two-state model or a sequential transition state model.

### Atomic force microscopy

AFM measurements were performed using a G5^1^–G5^7^ construct with two cysteine residues incorporated at the C terminus, to allow attachment to the gold-covered surfaces via gold-thiol covalent attachment. All AFM measurements were carried out in 20 mM Tris (pH 7.5), 150 mM NaCl, at 25 °C, using an Asylum Research MFP-3D microscope. Silicon nitride cantilevers with nominal spring constant of 30 pN nm^−1^ (Bruker MLCT) were used and calibrated using the thermal method^68^. One hundred-microlitre protein solution (250 μg ml^−1^ in AFM buffer) was adsorbed onto a gold surface and the AFM cantilever tip was used to pick it up by nonspecific adhesion, and then retracted at a constant speed (200, 800, 1,500, 3,000 and 5,000 nm s^−1^), measuring the force exerted by the protein in the process. Three independent experiments (different cantilevers and surfaces) were performed for each pulling speed. The unfolding force for all events from acceptable traces were measured and their force-extension profiles fitted to the worm-like chain model^69^ (with the persistence length fixed to 400 pm) using the IGOR Pro 6 software (WaveMetrics) to obtain Δ*L*_c_ values. The data from triplicates were pooled and the force and Δ*L*_c_ probability histograms were generated for each retraction rate. The modal force and Δ*L*_c_ values were calculated from Gaussian fits to the histograms using Mathematica 10 (Wolfram Research).

## Additional information

**Accession codes**: The atomic coordinates have been deposited in the Protein Data Bank under accession code number 4WVE. SAXS data and models have been deposited in the Small Angle Scattering Biological Data Bank www.sasbdb.org with codes SASDA37, SASDA47, SASDA57, SASDA67, SASDA77 and SASDA87.

**How to cite this article:** Gruszka, D. T. *et al.* Cooperative folding of intrinsically disordered domains drives assembly of a strong elongated protein. *Nat. Commun.* 6:7271 doi: 10.1038/ncomms8271 (2015).

## Supplementary Material

Supplementary Information Supplementary Figures 1-7, Supplementary Tables 1-2, Supplementary Discussion and Supplementary References

## Figures and Tables

**Figure 1:**
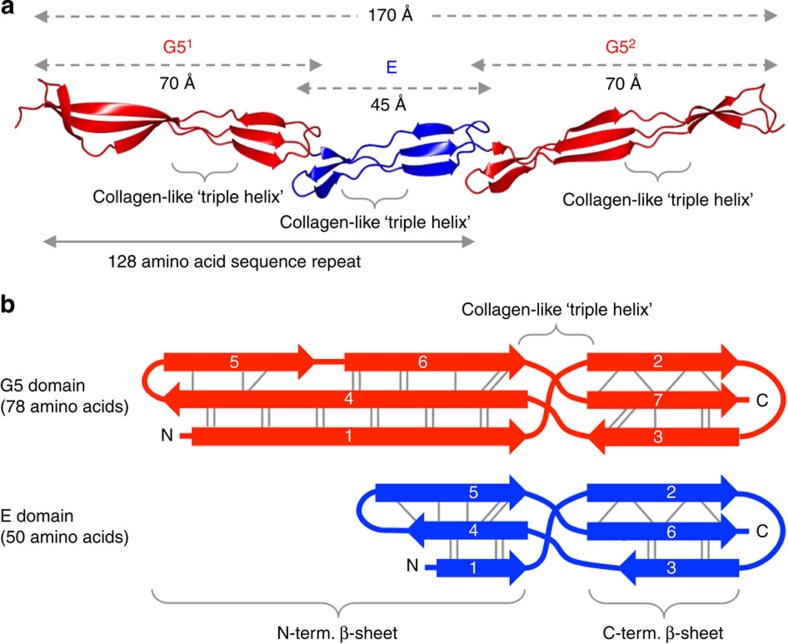
Overview of the SasG system. (**a**) Crystal structure of G5^1^-E-G5^2^ (PDB accession: 3TIQ) illustrating the head-to-tail arrangement of G5 (red) and E (blue) domains in SasG. The lengths of G5^1^-E-G5^2^ and individual domains, determined from the crystal structure, are shown. The 128 amino acid sequence repeat, covering a G5 and an E domain, is also indicated. (**b**) Schematic representation of the domain topology for G5 and E. The domains share the same fold (β-triple helix-β), defined by two single-layer, triple-stranded β-sheets connected via a central collagen-like triple-helical region (also indicated in **a**).

**Table 1:** Apparent equilibrium parameters for SasG domains.

	**G5**^**1**^**-WT**	**G5**^**1**^**-E-WT**	**G5**^**2**^**-WT**	**E-G5**^**2**^**-WT**	**E-G5**^**2**^**-T501C**^**A488**^**-E613C**^**A594**^	**E-G5**^**2**^**-E500W-E532C**^**IAEDANS**^	**G5**^**1**^**-E-G5**^**2**^**-WT**
*m*_D−N_ (kcal mol^−1^ M^−1^)	1.0±0.1	1.0±0.1	1.0±0.1	1.4±0.1	1.4±0.1	1.4±0.1	1.5±0.1
[urea]_50%_ (M)	3.2±0.1	3.2±0.1	2.8±0.1	4.4±0.1	4.3±0.1	4.3±0.1	4.9±0.1
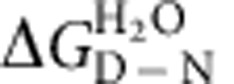 (kcal mol^−1^)	3.2±0.1	3.2±0.1	2.8±0.1	6.3±0.2	6.1±0.3	6.1±0.4	7.5±0.2

The parameters were obtained by fitting the data to a two-state model. The errors were calculated based on the errors of the fits.

**Table 2:** AFM parameters for SasG domains.

**Retraction rate (nm** **s**^**−1**^**)**	***n***		Force (pN)*		**Δ*****L***_**c**_ (Å)*	
	**E**	**G5**	**E**	**G5**	**E**	**G5**
200	126	52	212±25	351±34	149±15	219±9
800	409	146	250±35	421±36	145±12	216±6
1,500	597	229	229±27	379±28	154±10	224±8
3,000	258	149	254±35	397±53	152±12	224±13
5,000	547	263	265±37	408±35	153±13	227±9

AFM, atomic force microscopy.

Note that three independent experiments were performed at each pulling speed.

^*^Values (mode ± s.d.) were obtained from Gaussian fits to the histograms (see Supplementary Fig. 6).
