# Supplementary material for: Cooperative folding of intrinsically disordered domains drives assembly of a strong elongated protein
Source: Nat Commun. 2015 Jun 1;6:7271. doi: 10.1038/ncomms8271 (PMC4458895; doi:10.1038/ncomms8271)
Supplement: Supplementary Information — Supplementary Figures 1-7, Supplementary Tables 1-2, Supplementary Discussion and Supplementary References [file ncomms8271-s1.pdf]

Supplementary Figures

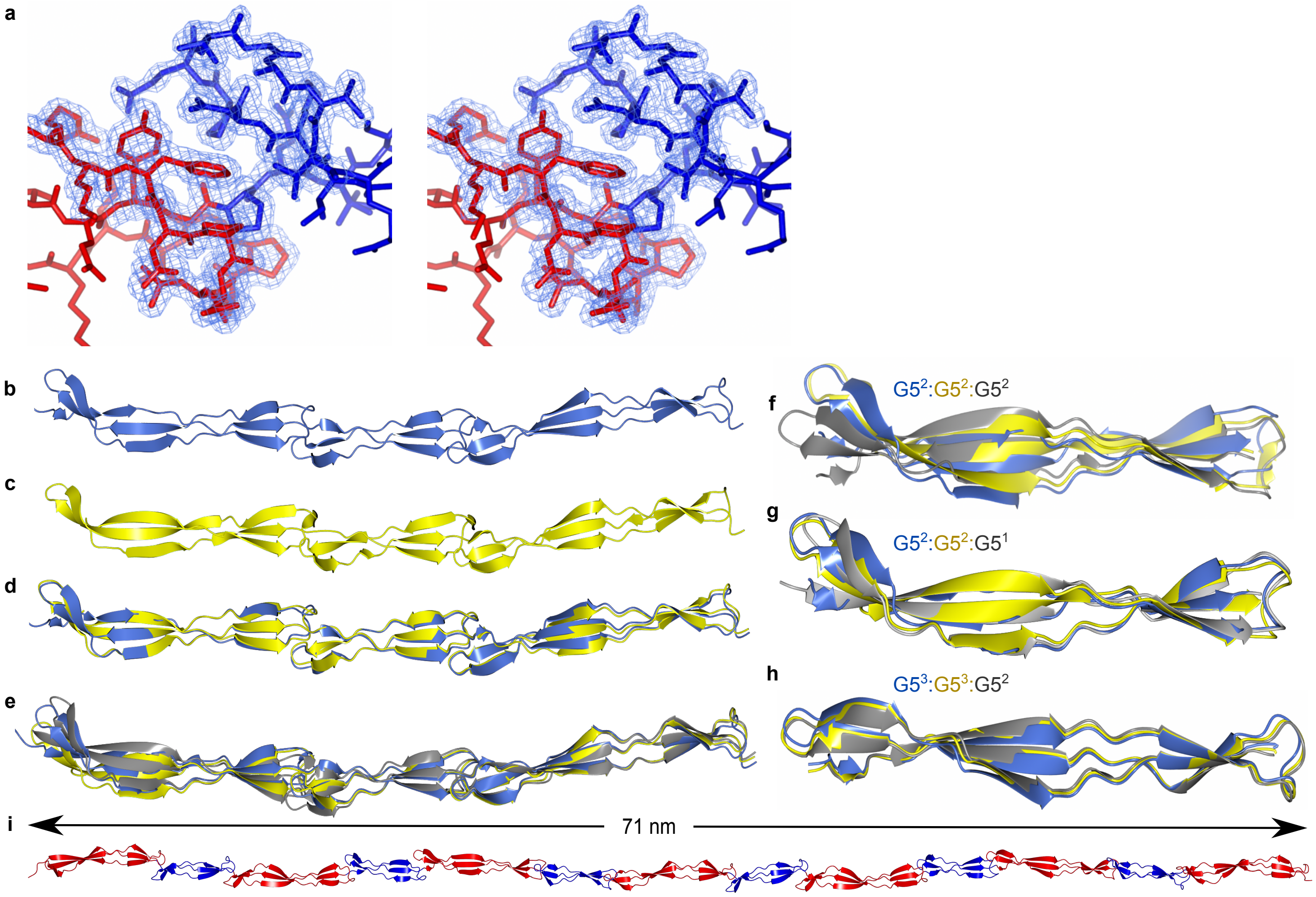

Supplementary Figure 1 | X-ray crystal structure of G5^{2}-G5^{3} and superposition with G5^{1}-G5^{2}. (**a**) Stereo image of the *2mFo-DFc* electron density map (blue) contoured at 1σ clipped to G5^{2}-G5^{3} residues at the interface of G5^{2} (red) and E^{2} (blue) (567–575; 625–628 and 657–665). X-ray crystal structure of G5^{2}-G5^{3} (**b**) chain A (blue) and (**c**) chain B (yellow). (**d**) Alignment of G5^{2}-G5^{3} chain A and chain B (Cα RMSD 1.2 Å, 194 residues aligned), and (**e**) aligned with G5^{1}-G5^{2} chain A (grey) (G5^{2}-G5^{3} chain A Cα RMSD 2.0 Å, 209 residues aligned; chain B Cα RMSD 1.6 Å, 184 residues aligned). (**f**) Alignment of G5^{2} from G5^{2}-G5^{3} (chains A and B) with G5^{2} from G5^{1}-G5^{2} chain A (chain A Cα RMSD 3.1 Å, 74 residues aligned; chain B Cα RMSD 2.3 Å, 67 residues aligned); and (**g**) with G5^{1} from G5^{1}-G5^{2} chain A (chain A Cα RMSD 1.5 Å, 77 residues aligned and chain B Cα RMSD 1.6 Å, 74 residues aligned). (**h**) Alignment of G5^{3} from G5^{2}-G5^{3} (chains A and B) with G5^{2} from G5^{1}-G5^{2} chain A (chain A Cα RMSD 1.4 Å, 78 residues aligned and chain B Cα RMSD 0.7 Å, 78 residues aligned). (**i**) A model of the repeating structure of SasG comprising repeats G5^{1}-G5^{7} generated by iterative superposition of X-ray crystal structures. The G5^{2}-G5^{3} structure and structure factor amplitudes were deposited in the PDB with accession code 4WVE.

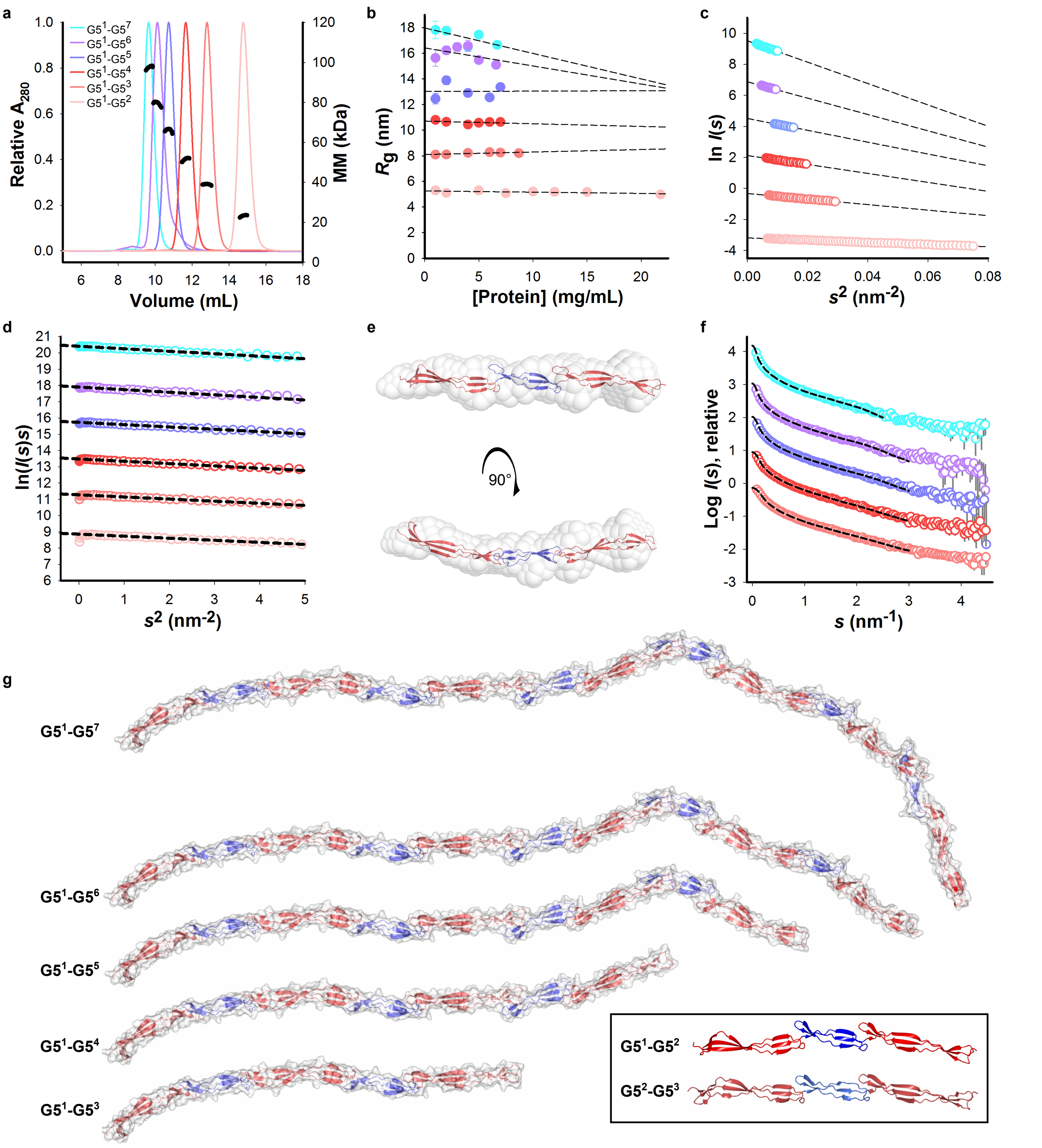

Supplementary Figure 2 │ Solution molecular mass and SAXS analysis of SasG. (a) Size exclusion chromatography multi-angle laser light scattering (SEC-MALLS) chromatograms (280 nm absorbance (left axis) and molecular mass (MM) (right axis)) of $G5^1$-$G5^2$, $G5^1$-$G5^3$, $G5^1$-$G5^4$, $G5^1$-$G5^5$, $G5^1$-$G5^6$ and $G5^1$-$G5^7$. (**b**) R_g calculated from $P(r)$ functions as a function of protein concentration (linear regression analysis, dashed lines). (**c**) Guinier plots (ln ($I(s)$ vs s^2) and (**d**) cross-sectional Guinier plots (ln ($I(s)s$) vs s^2) (scaled to construct length). (**e**) $G5^1$-$G5^2$ (PDB accession: 3TIQ) aligned to filtered average Gasbor model (Fig. 2b). (**f**) Calculated scattering (black dashed lines; see Supplementary Table 2 for χ^2 values) for rigid body models (**g**) generated from crystal structures $G5^1$-$G5^2$ and $G5^2$-$G5^3$ (boxed). Plots c, d, and f are offset on Log scale.

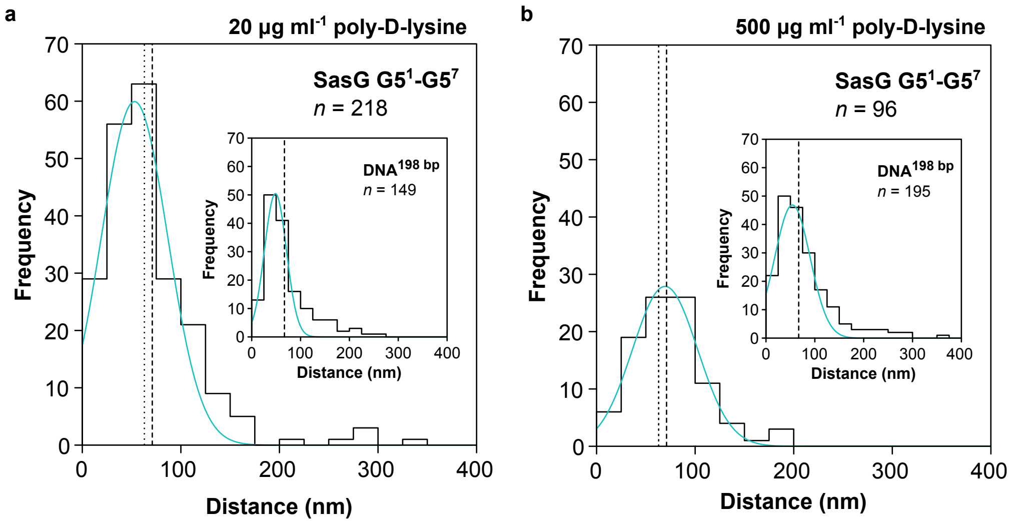

Supplementary Figure 3 │ SHRImP-TIRFM molecular end-to-end distance analysis of SasG. Inter-fluorophore distances for Alexa Fluor 488-labelled G5^1-G5^7 and 198 bp DNA (inset) on: (**a**) 20 µg ml^{-1} poly-D-lysine-treated quartz slide. Solid lines indicate Gaussian fits to the histograms (mean = 53 nm and 49 nm, respectively). (**b**) 500 µg ml^{-1} poly-D-lysine-treated quartz. Solid lines indicate Gaussian fits (mean = 69 nm and 54 nm, respectively). Dashed lines indicate the predicted end-to-end distances based on crystallographic data for G5^1-G5^7 and B-form DNA (71 nm (Supplementary Fig. 1i) and 67 nm, respectively) and dotted lines indicate the SAXS D_{max} of G5^1-G5^7 (63 nm).

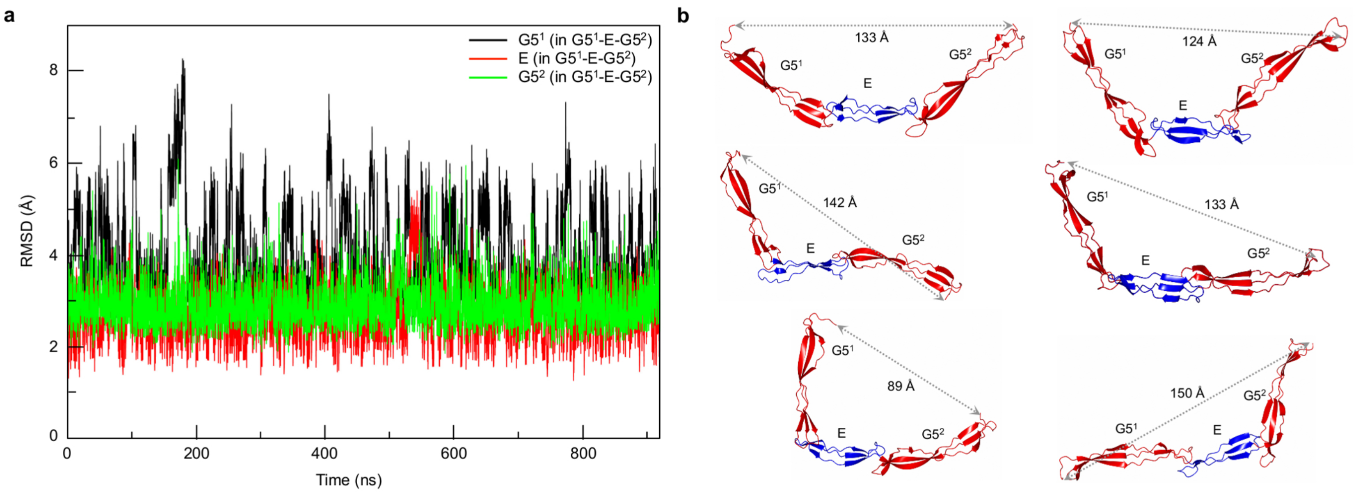

Supplementary Figure 4 | Conformational flexibility of G5^1-E-G5^2 probed by all-atom MD simulations. (**a**) A plot of RMSD against time for individual domains in the protein (G5^1 – black, E – red and G5^2 – green) confirms that all domains maintain their extended, native conformations throughout the simulation. (**b**) A subset of selected, transient, 'bent' conformations of G5^1-E-G5^2, associated with N-to-C distances shorter than 170 Å (extended conformation; Fig. 3c). Individual domains are extended, but their relative orientation changes over the course of the simulation, leading to reduced N-to-C distance. Such transient flexing occurs at G5-E and E-G5 interfaces. As the key inter-domain contacts are maintained during flexing, the structure quickly relaxes back to extended conformation (Fig. 3c).

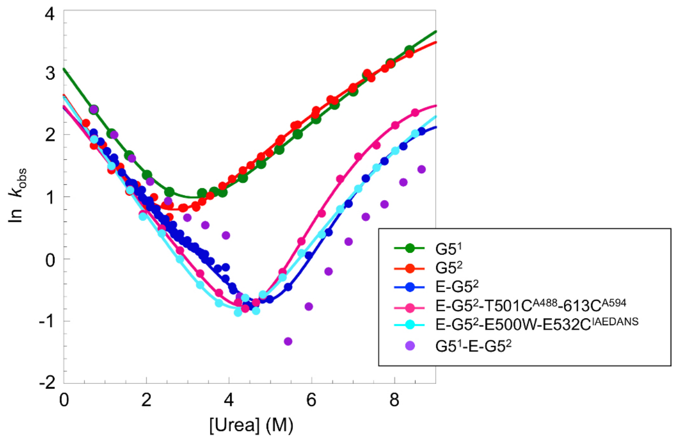

Supplementary Figure 5 │ Kinetics of SasG domains. Chevron plots (the dependence of the observed rate constants for folding and unfolding against the urea concentration) for wild type $G5^1$ (green), $G5^2$ (red), E-$G5^2$ (blue) and $G5^1$-E-$G5^2$, as well as E-$G5^2$-T501C^{A488}-E613C^{A594} (magenta) and E-$G5^2$-E500W-E532$C^{IAEDANS}$ (cyan).

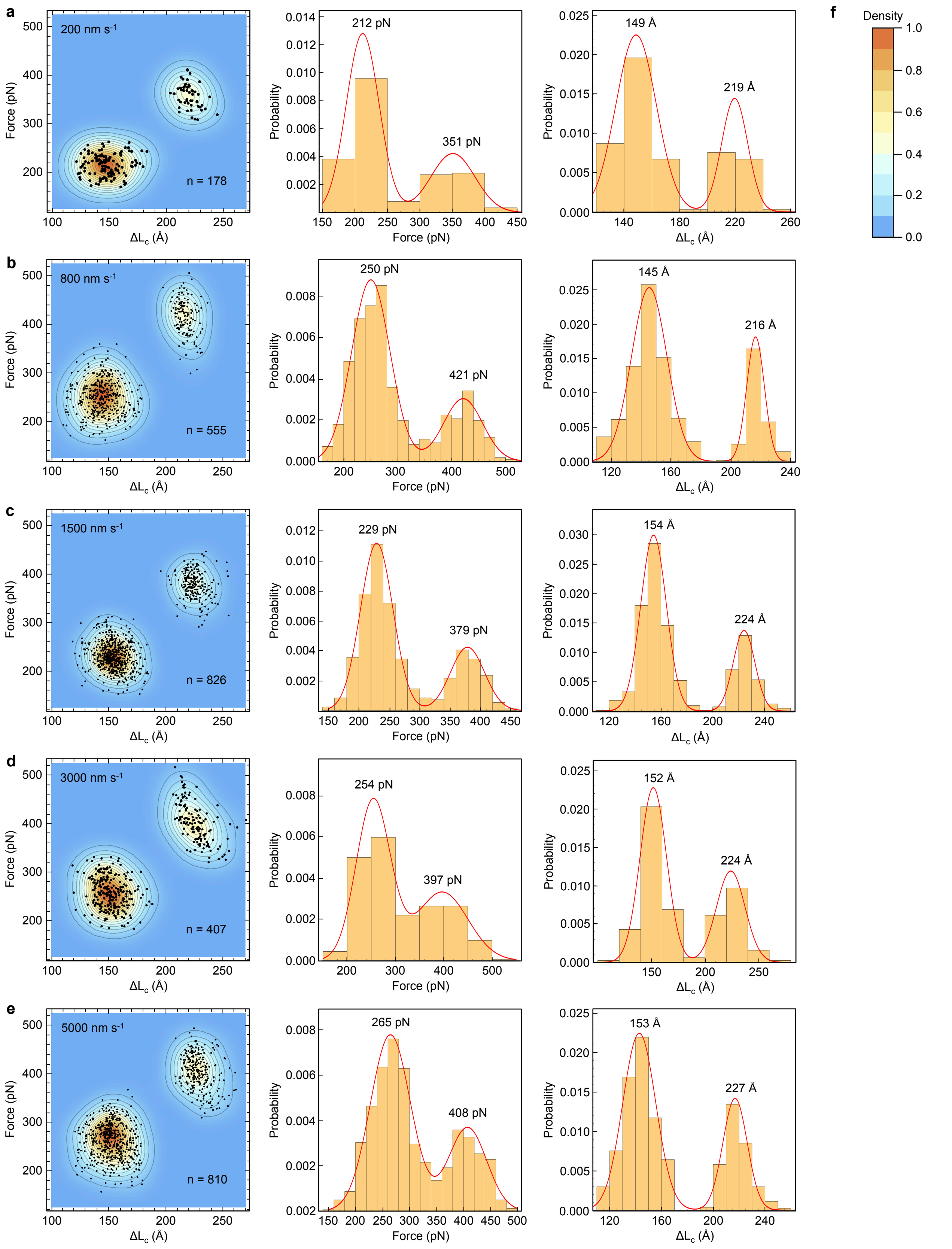

Supplementary Figure 6 │ Forced unfolding of SasG probed by AFM. Data were collected in triplicates (different surfaces and cantilevers) for a SasG construct containing seven G5 and six E domains ($G5^1$-$G5^7$) at the retraction rate of 200 nm s^{-1} (**a**), 800 nm s^{-1} (**b**), 1500 nm s^{-1} (**c**), 3000 nm s^{-1} (**d**) and 5000 nm s^{-1} (**e**). Data from triplicates were pooled and analyzed as described in the methods section. For each retraction rate a scatter plot superposed with a smooth density histogram is presented (left panel). The density histogram legend is shown in panel **f**. The corresponding force-frequency (central panel) and contour length gain (ΔL_c) - frequency (right panel) one-dimensional histograms are also shown for each retraction rate. Red lines indicate Gaussian fits to the histograms and the modal force and ΔL_c values are shown.

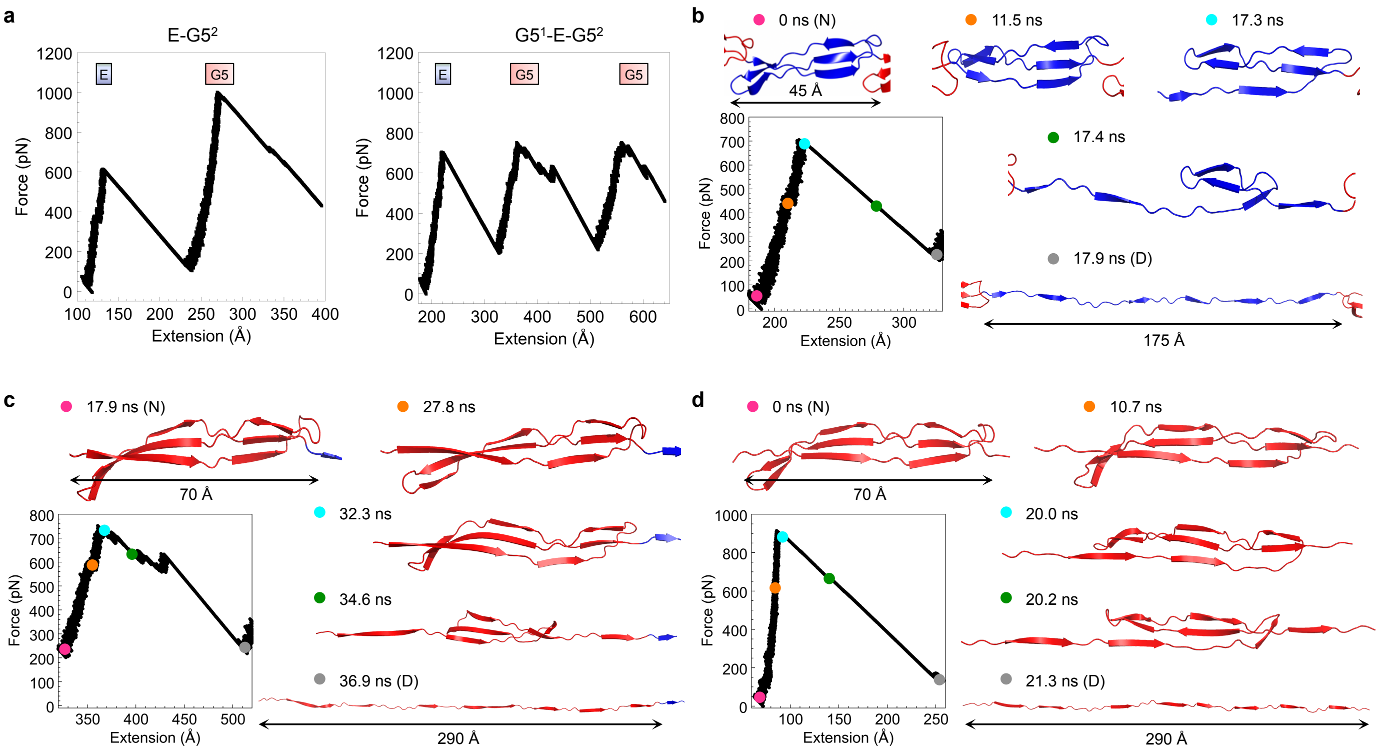

Supplementary Figure 7 | Forced unfolding of SasG domains probed by MD simulations. (**a**) Unfolding trajectories of E-G5^2 (left panel; PDB accession: 3TIP) and G5^1-E-G5^2 (right panel; PDB accession: 3TIQ). (**b**) Forced unfolding mechanism of the E domain in the context of G5^1-E-G5^2. (**c**) Forced unfolding mechanism of the G5^1 domain in the context of G5^1-E-G5^2. (**d**) Forced unfolding mechanism of the G5^2 domain in isolation. The figures show snapshots from unfolding simulations at 300 K, 5 pN nm^{-1}, 0.01 nm ps^{-1}. The snapshots are color-coded and their position on the corresponding unfolding trajectory is indicated. Native (N) and denatured (D) states are labeled and the measured N-to-C distances are shown.

Supplementary Tables

Supplementary Table 1 │Data collection and refinement statistics

**Data collection**	
Space group	*P*1
Cell dimensions	
a, *b*, *c* (Å)	39.9, 45.6, 60.4
α, β, γ (°)	84.8, 83.2, 78.6
Resolution (Å)	59.8-1.6 (1.63-1.60) *
R_{merge} (%)	7.7 (47.9)
$I/\sigma I$	15.8 (4.6)
Completeness (%)	98.9 (97.9)
Redundancy	4.2 (4.3)
Wilson B factor (Å^2)	14.4
**Refinement**	
Resolution (Å)	59.8-1.6
No. reflections	50959
$R_{work/}$ R_{free} (%)	20.7/24.9
No. atoms	
Protein	3243
Chloride	1
Water	257
B-factors	
Protein	25.9
Chloride	16.5
Water	31.5
R.m.s deviations	
Bond lengths (Å)	0.014
Bond angles (º)	1.73

*Highest resolution shell is shown in parenthesis.

Supplementary Table 2 │SAXS structural parameters and molecular mass determination

	G5[1]-G5[2]	G5[1]-G5[3]	G5[1]-G5[4]	G5[1]-G5[5]	G5[1]-G5[6]	G5[1]-G5[7]
**Structural parameters**						
I(*0*) (10^{-2} cm^{-1} mg^{-1}) (from *P*(*r*))	1.60±0.01	2.84±0.01	3.35±0.02	3.92±0.04	5.15±0.06	5.70±0.07
R_g (nm) (from *P*(*r*))	5.08±0.03	8.26±0.07	10.64±0.08	13.37±0.15	15.48±0.23	17.45±0.26
Guinier region sR_g range	0.20–1.11	0.45–1.12	0.51–1.10	0.73–1.10	0.71–1.12	0.63–1.09
R_g (nm) (from Guinier)	4.72±0.04	7.70±0.09	9.72±0.17	12.00±0.40	14.10±0.59	15.90±0.65
R_h (nm) (from QELS)	3.20±0.03	4.80±0.03	5.80±0.04	7.20±0.05	8.30±0.06	9.30±0.06
Shape Factor ($R_{g\ P(r)}/R_h$)	1.6	1.7	1.8	1.9	1.9	1.9
R_c (nm) (from modified Guinier)	0.51±0.01	0.53±0.01	0.55±0.01	0.57±0.01	0.58±0.01	0.58±0.01
D_{max} (nm) (from *P*(*r*))	19.0	30.5	38.5	48.0	57.0	63.0
D_c (nm) (from $P_c(r)$)	2.05	2.10	2.10	2.10	2.15	2.30
Porod Exponent (d_f)	1.04	1.04	1.08	1.06	1.07	1.10
Gasbor model χ^2 fit	1.12	0.96	0.92	1.08	1.02	0.77
Gasbor model NSD	1.35	1.76	2.36	3.32	4.04	4.58
Rigid body model χ^2 fit	-	1.19	0.94	1.13	1.15	1.20
**Molecular mass determination**						
Protein concentration (mg ml^{-1})	7.5	7.0	6.0	7.0	4.0	5.0
Partial specific volume (cm^3 g^{-1})	0.738	0.738	0.737	0.737	0.736	0.736
Contrast $\Delta\rho \times 10^{10}$ (cm^{-2})	2.923	2.929	2.935	2.942	2.944	2.947
MM (from *I*(*0*) from *P*(*r*)) (kDa)	20.7	36.6	43.1	50.3	66.0	72.9
MM (method of Fischer et al.) (kDa)	23.7	40.9	47.9	72.3	74.0	101.2
MM (calibrated with BSA) (kDa)	21.6	38.4	45.4	53.1	69.7	77.1
MM (from SEC-MALLS) (kDa)	23.1±0.02	38.7±0.04	51.5±0.05	66.4±0.07	79.6±0.08	98.4±0.10
MM (from ESI-MS) (kDa)	23.7	38.7	52.8	66.9	80.9	95.0
MM (from sequence) (kDa)	23.7	38.8	52.8	66.9	80.9	95.0

I(*0*) is the extrapolated zero angle intensity of scattering. R_g is the particle radius of gyration. R_c is the radius of gyration of a cross-section. D_{max} is the maximum linear particle dimension. D_c is the maximum dimension of a cross-section. Normalized spatial discrepancy (NSD) is a measure of similarity between three-dimensional SAXS *ab initio* models.

Supplementary Discussion

The structure of G5^{2}-G5^{3}

The Matthews coefficient[1] indicated that the asymmetric unit contained two molecules and had 46.9% solvent content, confirmed following phasing by molecular replacement (MR) (data statistics and refinement summarized in Supplementary Table 1). Relative to the near identical sequences of repeats G5^{2} – G5^{7}, G5^{1} is less conserved, with 85% sequence identity to G5^{2}. The X-ray crystal structures of G5^{2}-G5^{3} (Supplementary Fig. 1a-c) and G5^{1}-G5^{2} (PDB accession: 3TIQ) were superposed by secondary structure matching, revealing only modest divergence that was localized to the N-terminal G5 domain (Supplementary Fig. 1e). Superposition of G5^{2} alone from G5^{2}-G5^{3} chains A and B (residue ranges 549–626 and 552–626, respectively) showed some differences in the length of the β-strands, but overall these structures are highly similar (Cα root mean square deviation (RMSD) 1.3 Å, 74 residues aligned). When both structures were aligned with G5^{2} from G5^{1}-G5^{2} (3TIQ), deviation at the N-terminus of G5^{2}-G5^{3} was clear (Supplementary Fig. 1f). Comparison of G5^{2} from G5^{2}-G5^{3} with G5^{1} from G5^{1}-G5^{2} (residue range 420–498) showed that the N-terminal G5 domains share a common deviation from linearity (Supplementary Fig. 1g), suggesting that the presence of an E segment at the N-terminus of G5 may affect the folding of the G5 residues in the interface. To test this hypothesis, G5^{3} from G5^{2}-E^{2}-G5^{3} (residue range 676–754) and G5^{2} in G5^{1}-E^{1}-G5^{2} (having 97% sequence identity) were superposed, showing they are highly similar and linear (Supplementary Fig. 2h), supporting the role for E in maintaining the linearity of the C-terminal G5. To generate a model of the contiguous repeating unit, G5^{2}-G5^{3} and G5^{1}-G5^{2} were superposed by alignment of G5^{2}, then G5^{2}-G5^{3} was iteratively aligned to generate a model of the contiguous repeating unit 71 nm in length, comprising G5^{1}-G5^{7}, with 98.5% sequence identity to the wild-type sequence (Supplementary Fig. 1i).

SAXS analysis of the SasG repeat region

To investigate the solution structure of long contiguous repeats from SasG, we purified samples of G5^{1}-G5^{2}, G5^{1}-G5^{3}, G5^{1}-G5^{4}, G5^{1}-G5^{5}, G5^{1}-G5^{6}, and G5^{1}-G5^{7}. Their molecular masses (MM) were confirmed using electrospray ionization mass spectrometry (ESI-MS) (Supplementary Table 2). SEC-MALLS-QELS was used to analyze the homogeneity, monodispersity, solution MM and hydrodynamic radius (R_h) of purified proteins (Supplementary Table 2). All particles eluted as single peaks of MM close to that predicted from sequence analysis, with a uniform MM calculated across the peaks, indicating monodispersity (Supplementary Fig. 2a). The ratio of the weight average MM (M_w) to M_n (total mass/number of molecules) provides a measure of relative molar mass dispersity ($Đ_M$). In all cases, $Đ_M = 1$, confirming particles are monodisperse, and therefore suitable for SAXS analysis of particle shape. Initial analysis of SAXS scattering by the particles showed a slight concentration dependence of particle size, indicative of some interparticle repulsive effects at high concentration (Supplementary Fig. 2b). The highest concentration data set for each sample that had a minimal concentration dependent decrease in radius of gyration (R_g) was

selected for further analysis (Supplementary Table 2). The Guinier plots (ln $I(s)$ vs s^2) were linear at low angles ($sR_g \leq 1.1$), confirming monodispersity (Supplementary Fig. 2c). The R_g of these particles estimated by the Guinier approximation was consistently close, but slightly lower than those calculated from the distance distribution function ($P(r)$) (Supplementary Table 2). The Guinier region showed a progressive truncation with increasing construct size (Supplementary Fig. 2c), characteristic of scattering by highly anisotropic particles[2,3]. Calculation of $G5^1$-$G5^2$ and $G5^1$-$G5^3$ MM from absolute intensity ($I(0)$) derived from $P(r)$, calibrated to a BSA standard and using the method of Fischer *et al.*[4] agreed with the MM calculated from the sequence. The MM of $G5^1$-$G5^4$, $G5^1$-$G5^5$, $G5^1$-$G5^6$ and $G5^1$-$G5^7$ was underestimated by both absolute scattering and calibration to BSA, but the Fischer *et al.*[4] method defined MMs close to those expected (Supplementary Table 2).

SAXS scattering by all constructs was relatively featureless (Fig. 2e), which is a quality associated with highly anisotropic particle scattering[5]. $P(r)$ showed a clearly skewed distribution with maxima at short interatomic distances (Fig. 2c), also characteristic of scattering by rod-like particles[6]. We plotted a modified Guinier approximation (ln $I(s).s$ vs s^2) [2] which showed a maxima, followed by a linear correlation at higher angles, characteristic of rod-like particle scattering (Supplementary Fig. 2d) from which the radius of gyration of a cross-section (R_c) can be derived (Supplementary Table 2). The slight increase in R_c with increasing construct length suggests the rod may incorporate a slight curve or coil. $P(r)$ of a cross-section ($P_c(r)$) (Fig. 2d) shows a similar distance distribution for all particles, of 2.05–2.30 nm wide (Supplementary Table 2), correlating with the width of X-ray crystal structures of $G5^1$-$G5^2$ (PDB accession: 3TIQ) and $G5^2$-$G5^3$. Notably, R_c and $P_c(r)$ can also be calculated for a semi-flexible thread-like molecule and do not define particle rigidity[2], hence we calculated the slope of a line in the mid-*s* region of a Log $I(s)$ vs Log s plot, describing the Porod exponent. A Porod exponent of 1 defines a rigid one-dimensional particle in solution, which holds for all particles analyzed (Fig. 2f and Supplementary Table 2). Particle shape can also be assessed by determining the shape factor (ρ) (R_g/R_h), where ρ ~0.77 indicates a spherical particle, and ρ ≥1.73 a rod-like species. The shape factor of all particles falls in the range 1.6–1.9, again suggesting SasG repeats form contiguous rod-like shapes in solution (Supplementary Table 2). The length of an extended rod (l) can be derived from $l^2=12(R_g^2- R_c^2)$ [8], giving lengths of 17.5 nm, 28.5 nm, 36.8 nm, 46.2 nm, 53.5 nm and 60.4 nm for $G5^1$-$G5^2$, $G5^1$-$G5^3$, $G5^1$-$G5^4$, $G5^1$-$G5^5$, $G5^1$-$G5^6$ and $G5^1$-$G5^7$, respectively. To calculate the real space D_{max}, distance distribution functions ($P(r)$) were calculated for all particles (Fig. 2c). The particle lengths determined were similar to those estimated from the calculation described above (Supplementary Table 2). $P(r)$ functions were also used to derive R_g and $I(0)$ to overcome the limitations of Guinier analysis of these parameters due to the truncation of the Guinier region inherent for rod-like particle scattering (Supplementary Table 2). *Ab initio* modeling using Gasbor was repeated five times for all constructs and models were aligned and averaged (Fig. 2b; data fit shown Fig. 2e; model mean normalized spatial discrepancy (NSD) in Supplementary Table 2). The crystal structure of $G5^1$-$G5^2$ spatially aligns well with the filtered average shape derived from SAXS (Supplementary Fig. 2e). Rigid body modeling was performed using a modified version of SASREF[9] employing 50 spherical harmonics to take into account the

anisotropy of the particles. The calculation (total time, 2 weeks) was performed once in parallel against all datasets (Supplementary Fig. 2f,g). The rigid body models are consistent with the extended shapes from *ab initio* Gasbor modeling and describe highly extended rod-like structures that incorporate a slight coil/bend (goodness-of-fit χ^2 values[9] are listed in Supplementary Table 2, Fig. 2b and Supplementary Fig. 2f,g).

TIRF-microscopy analysis of particle length

To measure the macromolecular length, we analyzed Cys-G5[1]-G5[7]-Cys fluorescently labeled at N- and C-termini using SHRImP-TIRFM (Fig. 3a). Fluorophores attached to the protein were localized individually by sequential photobleaching, and inter-fluorophore distances were calculated to estimate the molecular end-to-end distance. Different concentrations of poly-D-lysine (20, 100 and 500 μg ml^{-1}) were used to treat the quartz microscope slides used for TIRFM, thus generating surfaces with an increasingly positive charge density. We obtained a range of end-to-end distances for G5[1]-G5[7] under these three conditions (mean ± s.e. = 59 ± 5 nm, Fig. 3b and Supplementary Fig. 3a,b). To validate our method under these conditions, DNA (198 bp) with a comparable inter-fluorophore distance was analyzed and gave a range of distances consistent with the adsorption of DNA to a positively charged surface (mean ± s.e. = 51 ± 2 nm, Fig. 3b inset and Supplementary Fig. 3a,b inset).

Both SasG and DNA are elongated macromolecules and negatively charged, thus it is appropriate to use polyelectrolyte theory to estimate their fractional surface charges in the low monovalent salt buffer (10 mM NaCl) utilized in the TIRFM experiments. According to the theory of Manning[10], a dimensionless parameter ξ (= β/b) can be calculated for a polyelectrolyte which relates the electrostatic interaction energy between adjacent charges in a polymer (Bjerrum length, β = 0.71 nm in pure water) to their average linear charge spacing (b). SasG has a larger charge spacing (predicted charge = -36 at pH 7 and L_c = 72.73 nm, thus b = 0.49 nm) compared to DNA (b = 0.17 nm) [10], thus it has a smaller ξ (≈ 1.4) than DNA (≈ 4.2) [10]. The fractional residual charge on the polyelectrolyte can be calculated as $(N\xi)^{-1}$, where N is the absolute value of the counterion valence (= 1 for Na^+ here). SasG and DNA are predicted to have fractional charges of 0.71 and 0.24, respectively (for $N = 1$). SasG retains more unscreened negative surface charges compared to DNA, because it condenses fewer counterions at its surface. As the valence of the counterion increases, *e.g.* for poly-D-lysine, SasG will retain a larger fractional residual charge relative to DNA. This will likely favor adsorption of SasG to a positively charged imaging surface in an equilibrated form indicative of the elongated, solution conformation – the mean end-to-end distances observed for SasG (Fig. 3b and Supplementary Fig. 3a,b) are consistent with this prediction. Our results also suggest that the G5[1]-G5[7] structure is intrinsically more rigid than double-stranded DNA of a comparable length under identical conditions (compare mean end-to-end distances in Fig. 3a and Supplementary Fig. 3a,b for DNA and SasG). For DNA, the mean end-to-end distances (49, 51 and 54 nm) were reduced at all imaging surface charge densities relative to the crystallographic B-form value (67 nm). This has been observed previously for DNA adsorbed on a positively charged surface[11] and is consistent with surface adsorption reducing the electrostatic tension in DNA due to charge neutralization, which in turn can reduce the apparent persistence

length, *i.e.* increasing the bending flexibility. The end-to-end distance distributions observed here for DNA (198 bp) are consistent with molecular equilibration on the imaging surface and a ~2-fold reduction in the apparent persistence length.

Thermodynamics of the SasG system

$G5^2$ in isolation has a free energy of folding ($\Delta G_{N\text{-}D}$) of −2.8 kcal mol^{-1} (from the equilibrium denaturation studies) whereas E-$G5^2$ is more stable, $\Delta G_{N\text{-}D}$ = −6.3 kcal mol^{-1}, a difference of 3.5 kcal mol^{-1} (Fig. 4 and Table 1). Hence, the folded E domain stabilizes $G5^2$ by 3.5 kcal mol^{-1}. However, we have demonstrated previously using NMR spectroscopy that the E domain is disordered in isolation[7]. If we make the conservative assumption that fewer than 15% of E molecules must be folded in isolation (or we would be able to detect them in the spectra) then the $\Delta G_{N\text{-}D}$ of E must be ≥ +1 kcal mol^{-1}. Thus, from our studies of $G5^2$ and E-$G5^2$, we estimate that the stability conferred by the E-$G5^2$ interface is *at least* 4.5 kcal mol^{-1}.

However, our studies of $G5^1$-E-$G5^2$, give us more information: $G5^1$ in isolation has $\Delta G_{N\text{-}D}$ = −3.2 kcal mol^{-1}, but it is stabilized in the context of $G5^1$-E-$G5^2$ (Fig. 4 and Table 1); the folded E domain stabilizes $G5^1$. From the equilibrium and kinetic studies of $G5^1$-E-$G5^2$ (Fig. 4, Supplementary Fig. 5 and Table 1) we can estimate $[\text{urea}]_{50\%}$ for $G5^1$ in the presence of E-$G5^2$ is ~ 4.7 M, which (since the *m*-value of $G5^1$ is 1 kcal mol^{-1}) allows us to estimate that $\Delta G_{N\text{-}D}$ of $G5^1$ is now approximately −4.7 kcal mol^{-1}. Thus, $G5^1$ is stabilized by the $G5^1$-E interface by ~1.5 kcal mol^{-1}.

Importantly, however, E is *unfolded*[7] in $G5^1$-E, so we can infer that the stability conferred by the $G5^1$-E interface (−1.5 kcal mol^{-1}) is insufficient to fold E. This allows us to put a minimal estimate on the free energy of folding of E as *at least* +2.5 kcal mol^{-1} (1 kcal mol^{-1} greater than the stability conferred by the interface, otherwise, folded E would be significantly populated at equilibrium in $G5^1$-E and would be detected in the NMR spectra[7]). Furthermore, we must revise our estimate of the stability conferred by the E-$G5^2$ interface, which not only enables E to fold (conferring *at least* −2.5 kcal mol^{-1}) but also stabilizes $G5^2$ (by −3.5 kcal mol^{-1}), i.e. the stability of the E-$G5^2$ interface is *at least* −6 kcal mol^{-1}.

Supplementary References

1 Matthews, B. W. Solvent content of protein crystals. *J. Mol. Biol.* **33**, 491-497 (1968).

2 Porod, G. Section I: The principles of diffraction, general theory. In *Small-Angle X-ray Scattering* (eds. Glatter, O. & Kratky, O.) 34-40 (Academic Press, London, UK, 1982).

3 Guinier, A. & Fournet, G. *Small-Angle Scattering of X-Rays*. (Wiley, New York, USA; Chapman and Hall, London, UK, 1955).

4 Fischer, H., Neto, M. D., Napolitano, H. B., Polikarpov, I. & Craievich, A. F. Determination of the molecular weight of proteins in solution from a single small-angle X-ray scattering measurement on a relative scale. *J. Appl. Crystallogr.* **43**, 101-109 (2010).

5 Feigin, L. A. & Svergun, D. I. *Structure analysis by small-angle X-ray and neutron scattering*. (Plenum Press, 1987).

6 Svergun, D. I. & Koch, M. H. J. Small-angle scattering studies of biological macromolecules in solution. *Rep. Prog. Phys.* **66**, 1735-1782 (2003).

7 Gruszka, D. T. *et al.* Staphylococcal biofilm-forming protein has a contiguous rod-like structure. *Proc. Natl Acad. Sci. USA* **109**, E1011-E1018 (2012).

8 Kratky, O. X-ray small angle scattering with substances of biological interest in diluted solutions. *Prog. Biophys. Mol. Bio.* **13**, 105-173 (1963).

9 Petoukhov, M. V. & Svergun, D. I. Global rigid body modeling of macromolecular complexes against small-angle scattering data. *Biophys. J.* **89**, 1237-1250 (2005).

10 Manning, G. S. The molecular theory of polyelectrolyte solutions with applications to the electrostatic properties of polynucleotides. *Q. Rev. Biophys.* **11**, 179-246 (1978).

11 Podestà, A., Indrieri, M., Brogioli, D., Manning, G. S., Milani, P., Guerra, R., Finzi, L. & Dunlap, D. Positively charged surfaces increase the flexibility of DNA. *Biophys. J.* **89**, 2558-2563 (2005).
